# On the Dynamics of Noncovalent Interactions Modulating Passive Diffusion of Cisplatin Across a Human Breast Cancer Cell Membrane: An amIGM + GLED View

**DOI:** 10.1002/cplu.70258

**Published:** 2026-10-13

**Authors:** Costantino Zazza, Daniele Belletto, Stefano Scoditti, Nico Sanna, Stefano Borocci, Emilia Sicilia

**Affiliations:** ^1^ Department for Innovation in Biological Agro‐Food and Forest Systems Università della Tuscia (DIBAF) Viterbo Italy; ^2^ Department of Chemistry and Chemical Technologies Università della Calabria Arcavacata di Rende Calabria Italy; ^3^ Donostia International Physics Center Donostia Spain; ^4^ Polimero eta Material Aurreratuak: Fisika, Kimika eta Teknologia Kimika Fakultatea Euskal Herriko Unibertsitatea UPV/EHU Donostia‐San Sebastian Spain; ^5^ CNR‐ISTP (Istituto per la Scienza e Tecnologia dei Plasmi) Bari Italy; ^6^ Istituto per i Sistemi Biologici del CNR (ISB) Sede di Roma ‐ Meccanismi di Reazione c/o Dipartimento di Chimica Sapienza Università di Roma Rome Italy

**Keywords:** breast cancer, metallodrugs, molecular dynamics, QTAIM, supramolecular chemistry

## Abstract

Passive permeation across biological membranes is governed by a delicate balance of transient noncovalent interactions that are difficult to resolve experimentally or through conventional molecular dynamics (MD) analyses alone. Here, we present a perspective on the integration of the averaged modified independent gradient model (amIGM) based on promolecular density approximation and the graphic representation of the local electron energy density (GLED) with classical MD simulations to investigate the passive translocation of the cisplatin (cisPt) anticancer drug across a model membrane representative of the lipid composition of breast cancer cells. This multiscale framework combines atomistic sampling with real‐space descriptors, enabling the dynamic characterization of hydrogen bonding, dispersion forces, steric effects, and local electronic stabilization trends along the permeation pathway. As a proof of concept, we analyze the supramolecular nanosolvation environment of cisPt at finite temperature in two representative states: bulk aqueous solution and membrane center, corresponding to the maximum of the potential of mean force (+16 kcal mol^−1^). The combined amIGM/GLED approach reveals how the nature and spatial distribution of drug–water interactions evolve in response to the local membrane environment, providing mechanistic insight into the electronic and supramolecular factors that shape the free‐energy landscape of passive permeation.

## Graphical abstract

A combined amIGM/GLED framework based on classical MD simulations at finite‐temperature conditions is proposed to investigate the passive permeation of cisplatin anticancer drug across a breast cancer model membrane. Dynamic real‐space analysis of nanosolvation reveals how noncovalent interaction patterns evolve from bulk water to the membrane free‐energy barrier.

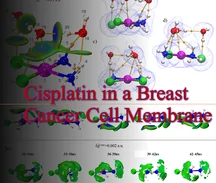

## 1 Introduction and Research Context

Cisplatin remains one of the most widely used chemotherapeutic agents in the treatment of breast cancer, particularly in triple‐negative breast cancer (TNBC), where the absence of the hormone receptor and HER2 expression actually limits the availability of targeted therapies [1, 2, 3, 4]. Its cytotoxic mechanism relies on covalent binding to nuclear DNA, forming intrastrand and interstrand crosslinks that trigger apoptotic pathways in rapidly dividing cells. However, the clinical efficacy of cisplatin is impaired not only by its specific intracellular reactivity but also by the efficiency with which it crosses the plasma membrane to reach its cytosolic and nuclear targets [5, 6]. For many years, cisplatin uptake was assumed to occur predominantly through passive diffusion, given the drug’s small size and net neutral charge [7, 8]. More recent evidence, however, has demonstrated that active and facilitated transport mechanisms—notably via the copper transporter CTR1 and organic cation transporters (OCTs)—contribute substantially to intracellular accumulation, particularly in breast tumor cell lines [9, 10]. Nonetheless, passive permeation across the lipid bilayer continues to represent a relevant and, in several contexts, rate‐limiting route of entry, especially in cells where transporter expression is downregulated as a resistance mechanism [11]. Reduced drug accumulation, largely attributable to impaired uptake and enhanced efflux, is indeed one of the principal determinants of acquired cisplatin resistance in breast cancer, underscoring the need for a molecular‐level understanding of how the drug interacts with and traverses the membrane independent of protein‐mediated transport [12, 13]. Moreover, the lipid composition of breast cancer cell membranes differs measurably from that of the healthy mammary tissue, with alterations in the cholesterol content, phospholipid headgroup distribution, and membrane fluidity that can significantly influence the thermodynamics and kinetics of small‐molecule permeation [14, 15].

Molecular dynamics (MD) simulations [16], combined with free energy methods such as umbrella sampling and potential of mean force (PMF) calculations [17], offer a powerful framework for dissecting the passive permeation pathway at atomistic resolution, capturing features such as the energetic barrier associated with the hydrophobic core of the bilayer, the role of headgroup interactions in the adsorption step, and the influence of membrane heterogeneity on diffusion rates. Such computational approaches complement experimental uptake assays by providing mechanistic insight that is difficult to obtain through bulk measurements alone [18, 19]. In this context, the averaged modified independent gradient model (amIGM) [20] based on promolecular density approximation offers a complementary perspective by enabling a real‐space quantitative characterization of noncovalent interactions—including van der Waals contacts, hydrogen bonding, and steric repulsion—directly from MD trajectories. This time‐resolved perspective is particularly valuable for condensed‐phase systems, where noncovalent contacts are transient, cooperative, and highly sensitive to the local chemical environment. Applied to biological membranes, amIGM‐based analysis can therefore help in disentangling the specific contributions—headgroup electrostatics, lipid tail dispersion forces, and solvent‐mediated interactions—that collectively shape the free energy landscape governing passive permeation. In this context, the graphical representation of the Local electron energy density *H*(*r*) (GLED) [21, 22] provides a real‐space visualization of regions where the total electronic energy density is locally stabilizing or destabilizing, thereby establishing a direct connection between the topology of the electron density and the covalent or noncovalent nature of chemical interactions [23]. In this perspective contribution, we highlight the potential of an integrated amIGM + GLED approach, coupled with classical MD simulations, for the investigation of the passive translocation of cisPt drug across a recently proposed model membrane representative of a breast cancer cell lipid environment; the composition of lipids and ions included in the membrane model is reported in Table 1 of Ref. [24]. In particular, given the complexity of the target system, we focused on the dynamics of supramolecular interactions at finite temperature, with particular emphasis on the nanosolvation patterns established between the cisPt metallodrug and surrounding water molecules. These interactions were characterized at two representative locations along the permeation pathway: the bulk aqueous phase and the midpoint of the membrane model, which corresponds to the maximum of the permeation free‐energy profile [25].

## 2 The amIGM + GLED Integrated Modeling

A direct investigation of the mutual geometrical contacts characterizing the passive permeation of the cisPt metallodrug across a human breast cancer membrane model is reported in Ref. [25] From this first computational investigation, an intriguing small water pocket encircling the drug molecule, which persists within the bilayer, is observed; cisPt is then capable of dragging water molecules into the bilayer core, locally reducing its hydrophobic character, as confirmed by the detection of water hydrogen bonds. In this respect, in Figure 1 (upper panel), we report, for the first time, an amIGM plot with two *δ* *ḡ* ^inter^ isovalues having the target of deriving—on average—the reservoir of cisPt/water supramolecular interactions sampled along a 60 ns production Classical MD simulation. In this respect, the drug and the water molecules are treated as two separate fragments, and a snapshot every 30 ps was used (1000 MD frames for the last 30 ns). Notwithstanding the local presence of only three molecules in the central part of the membrane, these are seen to be strongly confined in proximity to the cisPt while maintaining a certain degree of flexibility along the samplingroute [25]. Such a confinement mainly reflects the contextual presence of H‐bonding contacts between the hydrogen atoms (Hw) of water molecules and the Cl^−^ ligands and between the oxygen atom (Ow) and the NH_3_ moieties. Interestingly, the Pt(II) center also tends to coordinate water molecules in its first coordination shell, as remarked by the increase of the *δḡ* ^inter^ isovalue (see Figure 1, upper panel) in the associated amIGM plot. In the lower panel of the same figure, we also report the water‐density isosurfaces derived during the MD sampling. In agreement with the amIGM plots, water molecules are observed to fluctuate in the vicinity of the metallodrug, occupying both axial regions at $d_{H_{2}O}$ = 0.02 Å^−3^. At the higher isovalue, $d_{H_{2}O}$ = 0.04 Å^−3^, water molecules preferentially occupy positions where they can simultaneously interact with both the Cl^−^ ligands and the Pt(II) center. Another preferential hydration basin is located between the oxygen atom of water (Ow) and the H atoms of the neutral NH_3_ ligands.

**FIGURE 1:**
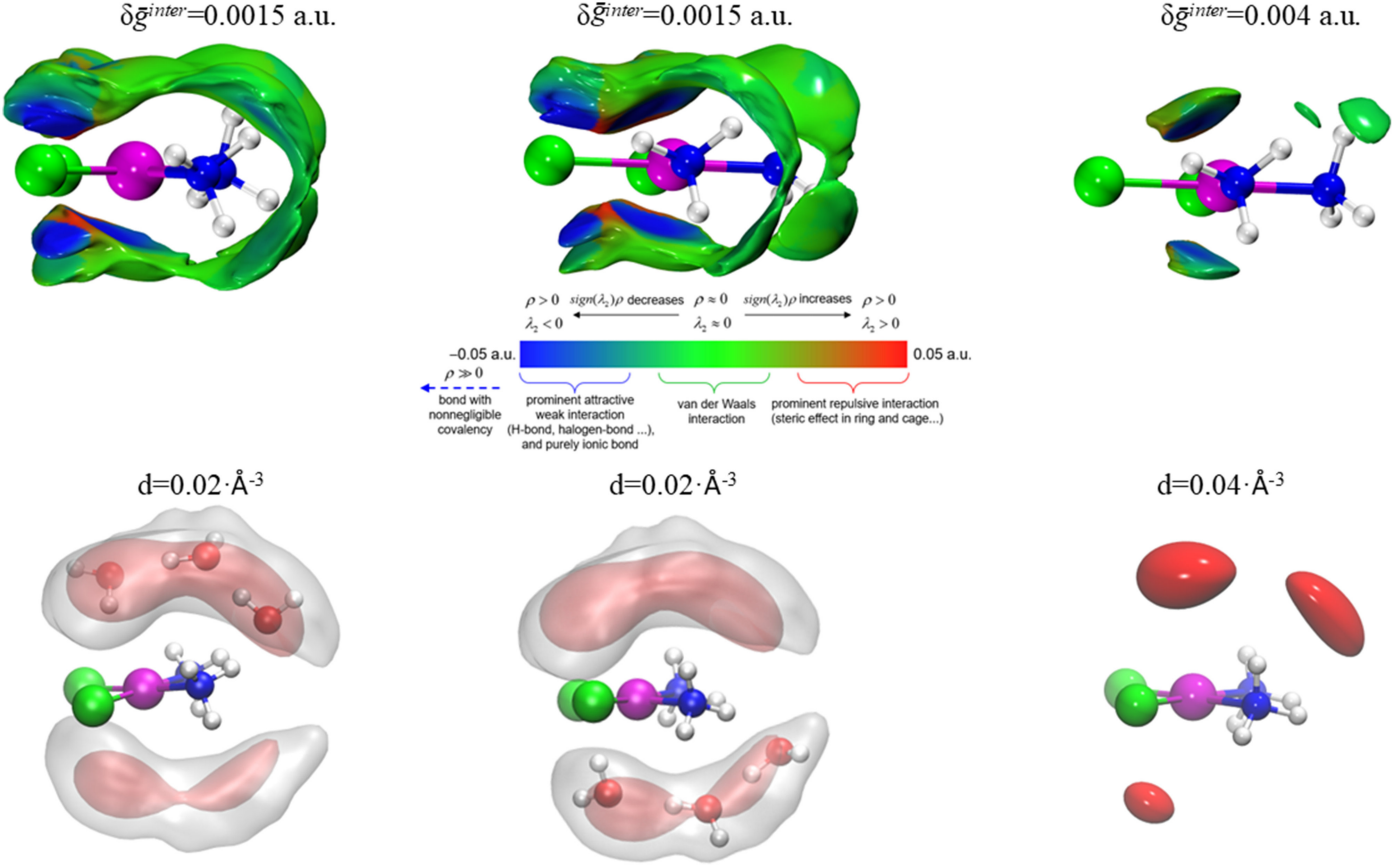
Upper panel: Sign(${\bar{\lambda }}_{2}$)$\bar{\rho }$ colored isosurfaces of *δ* *ḡ* ^inter^ = 0.0015 (two different orientations) and 0.004 a.u. corresponding to an amIGM analysis carried out considering 30 ns of MD simulation with the cisPt/wet drug in the middle point of a human breast cancer cell membrane model; a grid spacing of 0.12 *a*_0_ was used. Lower panel: water density isosurfaces (O in red, H in light gray) featuring solvent molecule distribution around the cisPt; two different isovalues of increasing value (0.02 and 0.04 Å^−3^) are used.

The dynamics of the noncovalent interactions are further studied by splitting the dynamics into ten different subtrajectories of 3 ns each (100 MD frames, from 30 to 60 ns) [25], and the results are reported in Figure 2. Looking at the estimated graphical representation of the mutual cisPt/water interactions via the amIGM algorithm, it is evident the dynamical behavior of the nanosolvation patterns in the membrane environment. In the majority of the sampled windows, the cisPt drug tends to assume a five‐fold‐like coordination shell, except for the 39–42 ns MD frames where a six‐fold coordination appears to be possible (see Figure 2a). This analysis is also performed, taking into consideration even all the phospholipids of the membrane model in contact with the investigated metallodrug within a cutoff of 5 Å. As a result, the amIGM representation shown in Figure 2b complements the analysis based solely on the local water molecules, providing a more comprehensive description of the three‐dimensional spatial distribution of the chemical interactions around the drug. In this specific case, the mutual interactions between the drug and the aliphatic regions of the phospholipids are represented via weak vdW interactions of variable nature featuring a light green sign(${\bar{\lambda }}_{2}$)$\bar{\rho }$ colored isosurfaces [20]. In turn, the computations are further extended, deriving the thermal fluctuation index (TFI) within the *δ* *ḡ* ^inter^ descriptor [26].^1^ TFI(amIGM) enables the interaction network to be interpreted not merely in terms of interaction strength but also according to its dynamical persistence. Inspection of the results reported in Figures S1 and S2 reveals that the dynamic lipid environment is seen to produce substantially greater fluctuations in the CisPt/membrane interaction network than those observed for the interfacial local water molecules. The dominant interaction regions are therefore expected to correspond primarily to NH···O_w_ H‐bonds between the ammine ligands and water molecules, followed by O_w_–H_w_···Cl^−^ interactions involving the coordinated chloride ligands; electrostatic interactions provide a further substantial contribution to the stabilization of the observed nanodroplet via Pt(IV)···O_w_ contacts connecting water molecules with the relatively electron‐deficient chelating site (see the blue regions in Figures 1 and 2a). Electrostatic interactions within the membrane media become significantly stronger, the first hydration shell becomes more structured, and the H‐bond network results in reinforcement. This trend is witnessed by a C‐PCM [30]/Cam‐B3LYP [31, 32] [D3(BJ)] [33, 34, 35] investigation on a selected MD frame. In this respect, the converged electronic wavefunction is used for estimating QTAIM‐based descriptors [36], IGMH [20, 26, see Endnote 1] isosurfaces, and for an investigation on the H‐Bond character via the GLED model [21, 22]. As can be clearly seen in Figure 3a, a strong and cooperative network of intermolecular interactions keeps the water molecules in close proximity to the cisPt drug. This analysis being carried out considering the water molecules as separated fragments also allows the characterization of the directionality and strength of the intramolecular H‐bonding contacts occurring in the cell membrane. We can appreciate a strong H‐bonding glue connecting H_w20_···O_w15_ and H_w16_···O_w12_ with (+3,−1) bond critical points (BCP, ***r*** **_cp_**) featuring *ρ*(***r*** **_cp_**) ~ 0.049 and *ρ*(***r*** **_cp_**) ~ 0.026 *e·a*_0_^−3^, respectively (see data in Table 1). It is worth noting that the promolecular‐density approximation employed to average the MD frames within the amIGM framework provides a suitable balance between the accuracy afforded by QM‐based calculations and the computational efficiency required for the direct analysis of classical MD trajectories, as further supported by the IGMH versus mIGM comparison reported in Figure S3.

**FIGURE 2:**
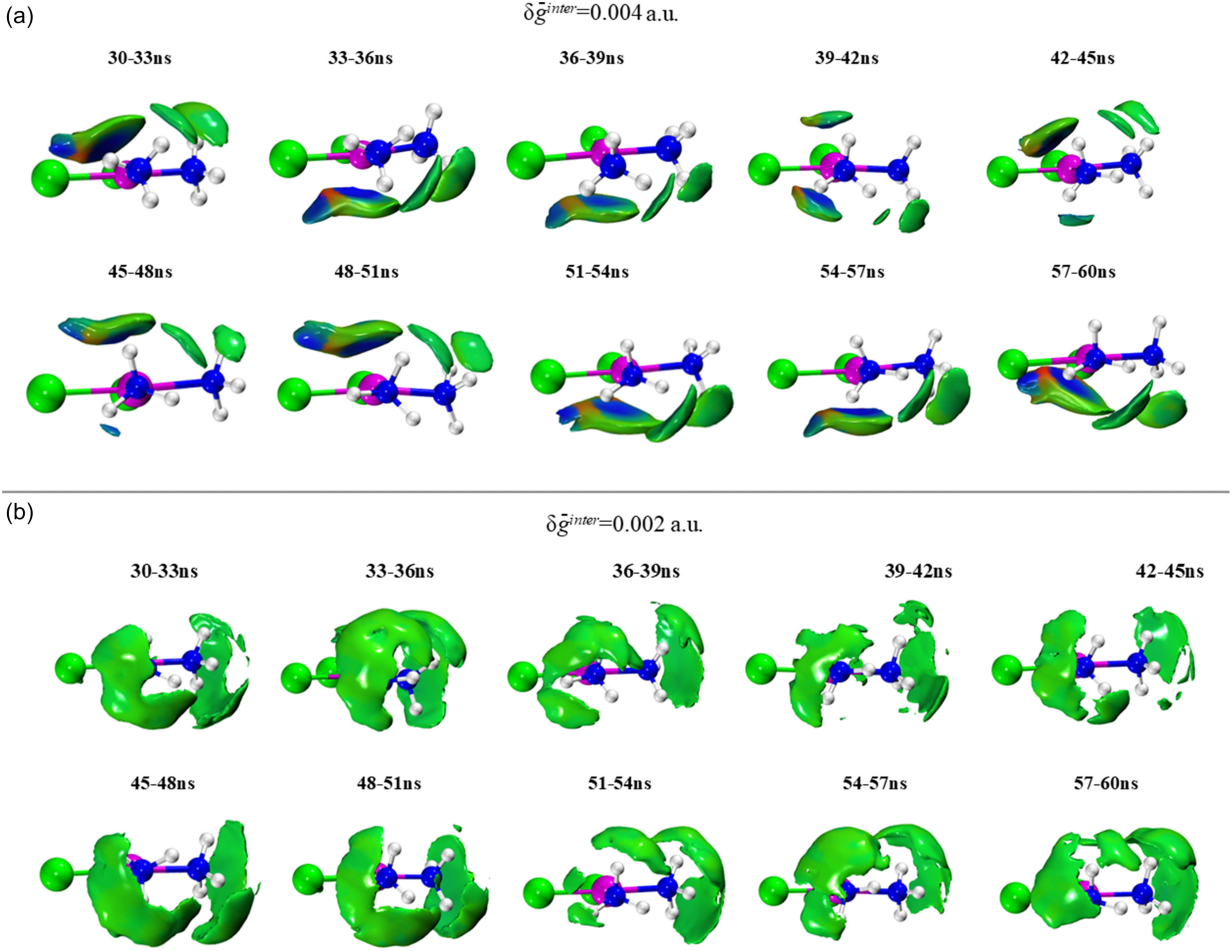
(a) Sign(${\bar{\lambda }}_{2}$)$\bar{\rho }$ colored isosurfaces of *δ* *ḡ* ^inter^ = 0.004 a.u. corresponding to an amIGM analysis with a grid spacing of 0.12 *a*_0_ out considering ten MD trajectories of 3 ns each with the cisPt/wet drug in the middle point of a human breast cancer cell membrane model; (b) the same as (a) but the amIGM analysis (*δ* *ḡ* ^inter^ = 0.002 a.u.) is performed considering all the phospholipids of the membrane model in contact with the cisPt drug. The color scale used is the same as that in Figure 1.

**FIGURE 3:**
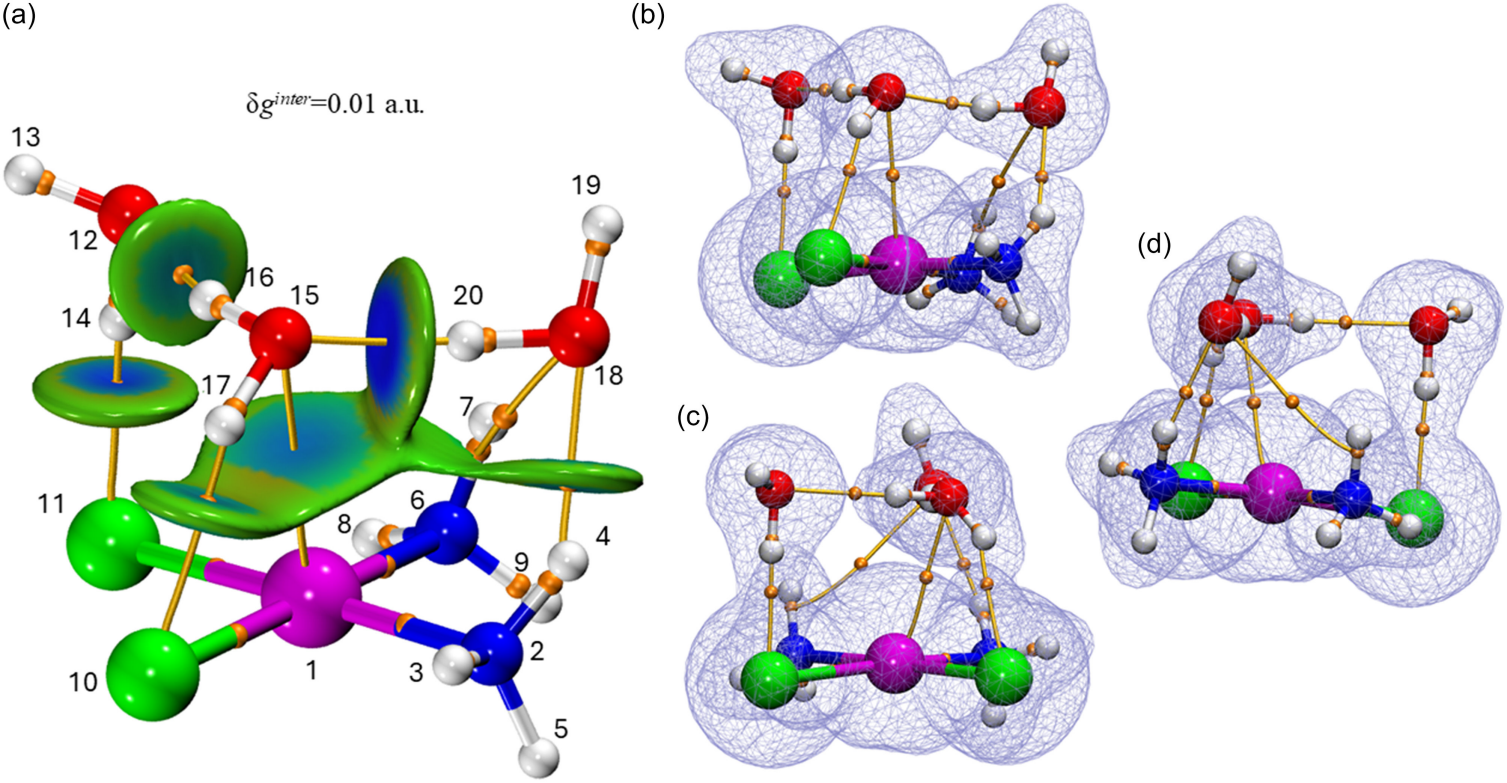
(a) (+3, −1) Bond critical points (BCPs) as derived from a QTAIM‐based analysis over an MD frame with the cisPt drug in the middle point of a human breast cancer cell membrane model. The derived IGMH plot is overimposed on the same structure. The color scale used is the same as that in Figure 1. All these analyses are performed at the C‐PCM/Cam‐B3LYP[D3(BJ)]/cc‐pVTZ level of theory. (b–d) Three different orientations showing the *H*(0, ISO) isosurface estimated at the same level of computation. A grid spacing of 0.12 *a*_0_ was used.

**TABLE 1:** Electron density *ρ*(*r*) (*e*·*a*_0_^−3^), Laplacian of electron density ∇^2^ *ρ*(*r*) (*e*·*a*_0_^−5^), electron kinetic energy density *G*(*r*) (hartree·*a*_0_^−3^), electron potential energy density *V*(*r*) (hartree·*a*_0_^−3^), and electron energy density *H*(*r*) (hartree·*a*_0_^−3^) for (+3, −1) BCPs [37, 38] on selected bonds of the investigated cisPt/H_2_O complex at C‐PCM/Cam‐B3LYP[D3(BJ)]/cc‐pVTZ level.

Connected atoms^a^	Distance, Å	*ρ*(***r***)	∇^2^ *ρ*(***r***)	*G*(***r***)	*V*(***r***)	*−G*(***r***)/*V*(***r***)	*H*(***r***)
15(O)–20(H)	1.66	0.049472	0.130735	0.043153	−0.053622	0.804763	−0.010469
11(Cl)–14(H)	2.01	0.037102	0.073416	0.025431	−0.032508	0.782301	−0.007077
4(H)–18(O)	1.91	0.026232	0.100184	0.023724	−0.022402	1.059012	0.001322
16(H)–12(O)	1.95	0.025934	0.071845	0.019114	−0.020266	0.943143	−0.001152
1(Pt)–15(O)	2.79	0.025492	0.060162	0.015287	−0.015565	0.982167	−0.000278
10(Cl)–17(H)	2.22	0.025003	0.067610	0.017683	−0.018464	0.957708	−0.000781
6(N)–18(O)	3.39	0.005815	0.019827	0.004048	−0.003140	1.289368	0.000909

a The atomic order is reported in Figure 3.

In particular, both these contacts present positive values of the ∇^2^ *ρ*(***r*** **_cp_**) coupled with negative values of the associated *H*(***r*** **_cp_**); this trend actually characterizes medium‐strong H‐bond pathways with a partially covalent nature [39, 40, 41, 42]. In the same figure, we also introduce (panel b–d) the results obtained by using the recently proposed GLED model. Such a method is seen to self‐consistently describe the chemical bond in terms of the three major categories typically used: the covalent bond, the ionic bond, and the noncovalent interactions driving the stability of more or less complex intermolecular aggregates. The major character of the bond (covalent or noncovalent) can be inferred by the visual inspection of the plotted *H*(***r***), particularly the 3D *H*(***r***) = 0 isosurface [i.e., *H*(0, ISO)]. When interacting moieties overlap their outermost *H* ^*+*^(***r***) regions, but their valence *H*(0, ISO) do not touch each other, and their enclosed valence *H* ^−^(***r***) contour lines remain well separated, the underlying contact features a noncovalent (nCov) character. On the other hand, when the two fragments overlap so as to produce an outer *H*(0, ISO) encompassing the nuclei of both fragments and enclosing the electrons with negative energies populating the valence *H* ^−^(***r***) binding domain (VBD) of their precursor moieties, the underlying interactions may be characterized as weakly covalent (wCov) [21, 22, 23, 42]. The *H*(0, ISO) displayed in Figure 3b–d let us suppose the presence of two different mutual interactions along the derived H_w_···O_w_ QTAIM Bond Paths (BPs). In fact, it can be seen that a wCov contact may be appreciated for the shortest H_w_···O_w_ contact lying at 1.68 Å; in contrast, the longer H_w_···O_w_ contact (1.95 Å) presents a (+3, −1) BCP (***r*** **_cp_**) located at the boundary between a wCov and a nCov(*t* = tight) interaction since the interacting fractions overlap their outermost *H* ^+^(***r***) regions, but their valence *H*(0, ISO) barely touch each other, leaving the enclosed valence *H* ^*−*^(***r***) regions slightly separated. In addition, these two contacts feature dissociation energies (*D*_e_) of 17.5_8_ and 9.6_9_ kcal/mol, respectively [21]. Moreover, it is interesting to highlight that another wCov contact may be observed between the H_w14_ and the Cl^−^_11_ moieties (see Figure 3d). A *ρ*(***r*** **_cp_**) ~ 0.037 *e·a*_0_^−3^ and a *H*(***r*** **_cp_**) of ca. −0.007 hartree*·a*_0_^−3^ are derived from QTAIM analysis as reported in Table 1. O_w18_ and N‐H_4_ moieties instead feature—over the ***r*** **_cp_** coordinates—a positive value of the *H*(***r*** **_cp_**), which indicates that this mutual intermolecular interaction is actually dominated by a local excess of kinetic energy [i.e., *G*(***r*** **_cp_**) >* V*(***r*** **_cp_**) and *−G*(***r*** **_cp_**)/*V*(***r*** **_cp_**) > 1, see Table 1 and Figure 3b]. The Pt(II)···O_w_ mutual interactions, given the *ρ*(***r*** **_cp_**) ~ 0.025 *e·a*_0_^−3^, the positive ∇^2^ *ρ*(***r*** **_cp_**) associated with a negative *H*(***r*** **_cp_**) (although close to zero), and the *H*(0, ISO) plot may be rationalized as nCov(t). The H_w17_···Cl^−^_10_ BP at 2.22 Å also features the same trend with similar *ρ*(***r*** **_cp_**), ∇^2^ *ρ*(***r*** **_cp_**), and *H*(***r*** **_cp_**). The weakest O_w18_···N_6_ interaction involves vdW forces (typically dispersion forces), which are present but generally much weaker than the other interactions previously described. At last, we would like to mention that the dielectric constant of *ε* = 4 adopted for the membrane interior in the C‐PCM calculations was selected to represent the relatively low‐polarity and low‐polarizability environment of the hydrophobic membrane core [43]. While a quantitative correlation between amIGM/GLED descriptors and the PMF would require a systematic analysis of a statistically representative ensemble of configurations sampled along the entire permeation pathway [25], the present analysis is intended to provide a complementary qualitative interpretation of the free‐energy profile by visualizing the local non–covalent interaction network at a representative stage of the permeation process.

When the hydration of cisPt in the bulk water is concerned, the water density isosurfaces derived reveal a rather different scenario with respect to that appreciated in the membrane. As a matter of fact, the water distribution is much more isotropic and is capable of encircling the entire structure of the solute (see Figure 4). Increasing the O_w_ density (from 0.02 to 0.04 Å^−3^), as expected, the Pt(II) cation when surrounded by water molecules promotes the formation of a six‐coordinate cisPt(II) hydration environment with two water molecules preferentially occupying the axial positions [44–46]. Having a look at the noncovalent interactions arising along the 30 ns of MD simulation, the amIGM plot in Figure 4 reveals the effects of nanosolvation patterns in terms of mutual drug/water interactions. A relatively small *δ* *ḡ* ^inter^ provides an amIGM isosurface that encloses the drug, while a larger value (0.004 a.u.) reveals a more punctual picture that shows the effective supramolecular interactions maintaining the water molecules in the Pt(II) axial site and encircling the two NH_3_ ligands.

**FIGURE 4:**
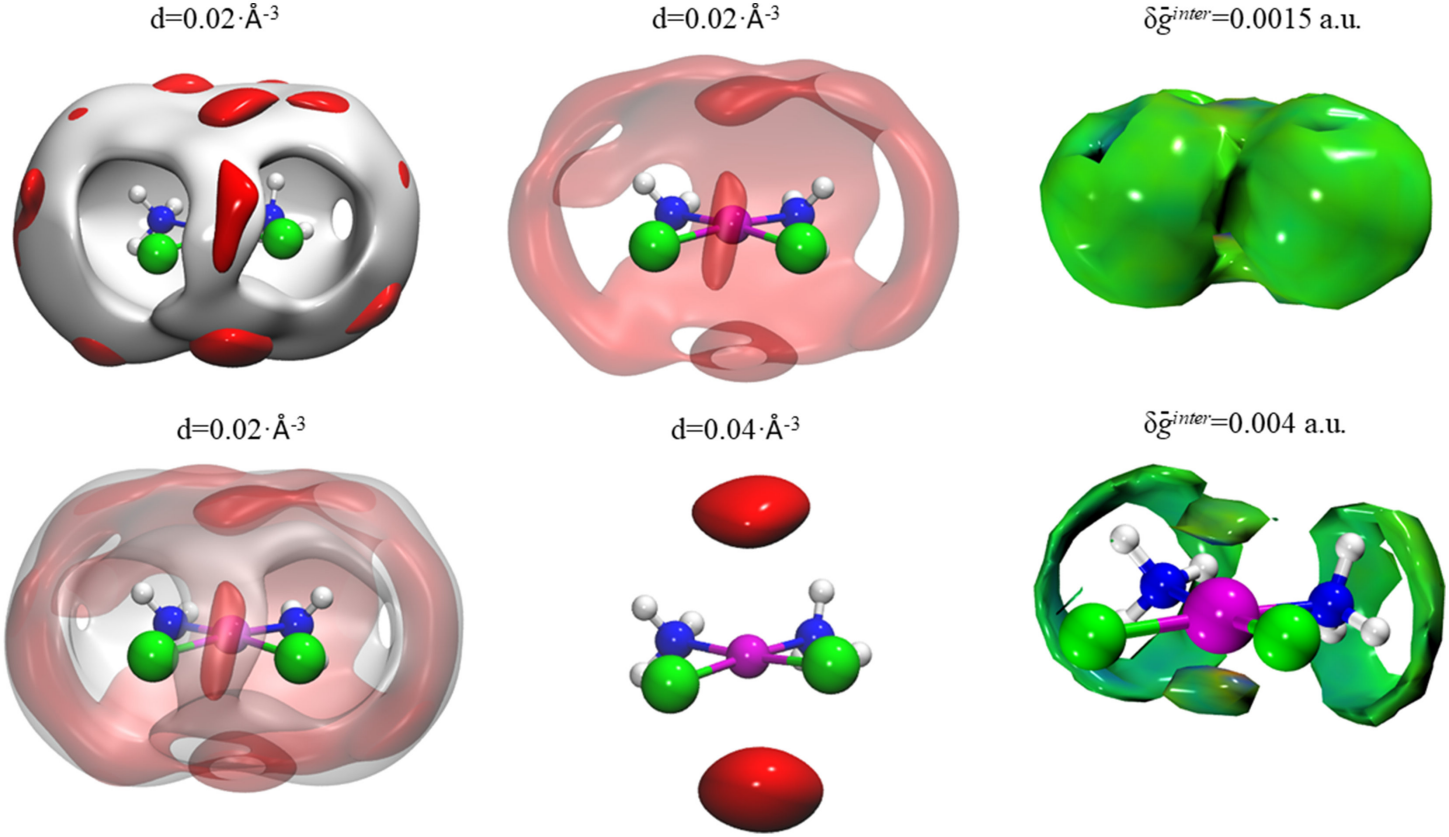
Water density isosurfaces (O in red, H in light gray) featuring solvent molecule distribution around the cisPt solute in bulk water environment; two different isovalues of increasing value (0.02 and 0.04 Å^−3^) are used. Sign(${\bar{\lambda }}_{2}$)$\bar{\rho }$ colored isosurfaces of *δ* *ḡ* ^inter^ = 0.0015 (two different orientations) and 0.004 a.u. corresponding to an amIGM analysis carried out considering 30 ns of MD simulation with a grid spacing of 0.12 *a*_0_. The color scale used is the same as that in Figure 1.

In conclusion, this perspective highlights how the combined amIGM + GLED framework opens new avenues for the rational investigation of the fluctuating chemical interactions that govern the nanosolvation pathways of the cisPt drug in complex biological environments, such as a model membrane of a human breast cancer cell under finite‐temperature conditions. With respect to the previous MD contribution [25], this investigation, although limited to two representative windows corresponding to cisPt in bulk water and in the central region of the membrane, provides a qualitative picture about the dynamics of the interactions modulating the mutual position of the drug in the different environments. Moreover, while this analysis does not aim to capture quantitatively the membrane‐translocation process, the comparison between the two representative states offers useful insights into the changes in the local interaction network experienced by cisPt upon moving from the aqueous phase to the hydrophobic membrane interior. Looking ahead, the generality and transferability of this framework make it broadly applicable to the investigation of dynamic chemical interactions in complex molecular systems, offering new opportunities to bridge atomistic simulations with quantitative descriptors of structure–dynamics–function relationships in chemistry, biology, and materials science.

## Disclaimer

The opinions expressed in this publication are the view of the author(s) and do not necessarily reflect the opinions or views of ChemBioChem, the Publisher, ChemistryEurope, or the affiliated editors.

## Funding

This study was supported by CINECA (CNHPC_1453641) and Spanish State Research Agency (JDC2023052228I).

## Conflicts of Interest

The authors declare no conflicts of interest.

## Supporting information

Supplementary Material

## Data Availability

The data that support the findings of this study are available from the corresponding author upon reasonable request.

## Endnotes

1 The independent gradient model (IGM) and independent gradient model based on Hirshfeld partition (IGMH) are closely related methods for visualizing and quantifying noncovalent interactions, but they differ fundamentally in how they define the underlying electron density. IGMH replaces the promolecular density with the actual electron density obtained from a quantum calculation. The Hirshfeld partitioning scheme applied defines a weighted atomic density:

(1)
$$\rho_{i}^{\text{Hirsh}}\left(r\right)=\rho \;\left(r\right)\cdot w_{i}\;(r)$$

where *ρ*(***r***) is the actual electron density while the Hirshfeld weighting function of the atom *i* is defined as:

(2)
$$w_{i}\;\left(r\right)=\frac{\rho_{i}^{0}\;(r)}{\rho^{0}\;(r)}=\frac{\rho_{i}^{0}\;(r)}{\sum_{j}^{\text{Natoms}}\rho_{j}^{0}\;(r)}$$

where index *j* loops over all atoms, and the *ρ* ^0^(***r***) in the denominator is a promolecular density, and *ρ*_*i*_^0^ stands for spherically averaged density around atom *i* in its unperturbed (free) state [20]. In the IGM model, the *ρ* ^0^(***r***) is defined on densities of atoms in their free states under the so‐called promolecular approximation. On the contrary, in the IGMH method, the free‐state atomic density (e.g*.*, *ρ*_*i*_^0^) in the IGM description is with *ρ*_*i*_^Hirsh^. The key descriptor of IGM‐based models is the *δg* ^inter^ function, which isolates intermolecular (or interfragment) interactions:

(3)
$$\delta g^{\text{inter}}=|\nabla \rho^{\mathrm{IGM}}\;(r)|-|\nabla \rho \;(r)|$$

where *ρ* ^IGM^(***r***) is the electron density constructed as the sum of the densities of isolated promolecular fragments:

(4)
$$\rho^{\mathrm{IGM}}\left(r\right)=\sum_{\mathrm{fragment}}\;\;\rho {\left(r\right)}_{\text{fragments}}$$

In the Hirshfeld‐based version, the function is modified to account—starting from a converged electronic wavefunction—for atomic partitioning as follows:

(5)
$$\delta g_{\text{Hirsh}}^{\text{inter}}=\sum_{i\epsilon \mathrm{frag}1\;}\;\sum_{j\epsilon \mathrm{frag}2}\;|\nabla \rho_{i}\;\left(r\right)|+|\nabla \rho_{j}\;\left(r\right)|-|\nabla \left(\rho_{i}\;\left(r\right)+\rho_{j}\;\left(r\right)\right)|$$

The principle is brilliant in its simplicity; if there were no interaction between the *i* (fragment 1) and *j* (fragment 2) couple of atoms, the additive property of gradients would apply:

(6)
$$g_{i\text{,}j}\;\;(r)\;\text{=}|\nabla \left(\rho_{i}\;\;\left(r\right)+\rho_{i}\;\;(r)\right)|=|\nabla \rho_{i}\;\;\left(r\right)|+|\nabla \rho_{j}\;\;(r)|=g_{i\text{,}j}^{\mathrm{IGM}}\;\left(r\right)$$

and therefore ${\delta g}_{\mathrm{Hirsh}}^{\mathrm{inter}}=0$; when interaction occurs, the electron density is redistributed and:

(7)
$$|\nabla \left(\rho_{i}\;\;(r)+\rho_{j}\;\;(r)\right)|\neq |\nabla \rho_{i\;}\;\left(r\right)|+|\nabla \rho_{j}\;\;(r)|$$

the difference measures how much the combined system deviates from the behavior of two isolated, noninteracting, atoms. In the IGMH analysis the $\rho_{i}$ terms over isolated fragments in the above expressions correspond to $\rho_{i}^{\text{Hirsh}}$. The $\delta g_{\text{Hirsh}}^{\text{inter}}$ related isosurfaces are then used to visually appreciate the interaction regions, while the function sign(*λ*_2_)*ρ*(***r***) is superimposed on the estimated isosurfaces to visually characterize the nature of the chemical interactions. The latter indicates the product of the electron density, *ρ*, and the sign of the second largest eigenvalue of the Hessian matrix of the electronic density; *ρ* is generally positively correlated with the interaction force, while sign(*λ*_2_) has instead the ability to distinguish whether the interactions are attractive or repulsive. Thus, interaction patterns are visualized in a 3D space by plotting a chosen low‐value isosurface of the $\delta g_{\text{Hirsh}}^{\text{inter}}$, colored by the sign(*λ*_2_)*ρ*(***r***). Here, we apply a variant of IGM, referred to as mIGM. The approach is conceptually simple: all terms are evaluated as in IGMH, except that the promolecular density is employed in place of the actual molecular density. Since mIGM, like IGM, relies exclusively on atomic coordinates, the computational cost remains essentially unchanged. Inspired by the aNCI approach [27, 28], the aIGM and amIGM methods are developed [29] to enable IGM and mIGM to investigate average weak interactions in dynamic environments. In these methods, interfragment interactions are visualized using isosurfaces of the averaged $\delta g^{\text{inter}}$($\delta {\bar{g}}^{\text{inter}}$), which is obtained by averaging the $\delta g^{\text{inter}}$ values calculated for all frames of a MD trajectory:

(8)
$$\delta {\bar{g}}^{\text{inter}}\left(r\right)=\frac{1}{N}\sum_{k=1}^{N}\;\delta g_{k}^{\text{inter}}\;\left(r\right)$$

where *N* corresponds to the number of MD frames selected, and the sign(${\bar{\lambda }}_{2}$)$\bar{\rho }$ is used to color the isosurfaces of $\delta {\bar{g}}^{\text{inter}}$, similar to the aNCI method [27]. In addition, to quantify the extent of thermal fluctuations affecting the interaction *δḡ*(***r***)^inter^ patterns identified along the MD time windows selected (see Figure 2), we also evaluated the associated TFI according to the following equation:

(9)
$$\mathrm{TFI}\;\left(r\right)=\frac{\sigma_{\delta g^{\text{inter}}\;(r)}}{\delta {\bar{g}}^{\text{inter}}\;(r)}=\frac{\sqrt{\frac{1}{N-1}\sum_{k=1}^{N}{\left[\delta g^{\text{inter}}\;\left(r\right)-\delta {\bar{g}}^{\text{inter}}\;(r)\right]}^{2}}}{\delta {\bar{g}}^{\text{inter}}\;\left(r\right)}$$

In this context, low TFI values indicate supramolecular interactions that remain comparatively stable throughout the trajectory, whereas larger values identify highly fluctuating contacts that are more sensitive to conformational and thermal effects.

## References

[1] R. T. Powell , A. L. Rinkenbaugh , L. Guo , et al., “Targeting Neddylation and Sumoylation in Chemoresistant Triple Negative Breast Cancer,” npj Breast Cancer 10 (2024): 37. 10.1038/s41523-024-00644-4 PMC11130334 38802426

[2] G. Bianchini , J. M. Balko , I. A. Mayer , M. E. Sanders , and L. Gianni , “Triple‐Negative Breast Cancer: Challenges and Opportunities of a Heterogeneous Disease,” Nature Reviews Clinical Oncology 13 (2016): 674. 10.1038/nrclinonc.2016.66 PMC5461122 27184417

[3] S. Ghosh , “Cisplatin: The First Metal Based Anticancer Drug,” Bioorganic Chemistry 88 (2019): 102925. 10.1016/j.bioorg.2019.102925 31003078

[4] N. Muhammad , M. Hanif , and P. Yang , “Beyond Cisplatin: New Frontiers in Metallodrugs for Hard‐to‐Treat Triple Negative Breast Cancer,” Coordination Chemistry Reviews 499 (2024): 215507.

[5] N. Martinho , T. C. B. Santos , H. F. Florindo , and L. C. Silva , “Cisplatin‐Membrane Interactions and Their Influence on Platinum Complexes Activity and Toxicity,” Frontiers in Physiology 9 (2019): 1898. 10.3389/fphys.2018.01898 PMC6336831 30687116

[6] S. Rottenberg , C. Disler , and P. Perego , “The Rediscovery of Platinum‐Based Cancer Therapy,” Nature Reviews Cancer 21 (2021): 37. 10.1038/s41568-020-00308-y 33128031

[7] H. Burger , W. J. Loos , K. Eechoute , J. Verweij , R. H. J. Mathijssen , and E. A. C. Wiemer , “Drug Transporters of Platinum‐Based Anticancer Agents and Their Clinical Significance,” Drug Resistance Updates 14 (2011): 22. 10.1016/j.drup.2010.12.002 21251871

[8] D. Sahoo , P. Deb , T. Basu , S. Bardhan , S. Patra , and P. K. Sukul , “Advancements in Platinum‐Based Anticancer Drug Development: A Comprehensive Review of Strategies, Discoveries, and Future Perspectives,” Bioorganic & Medicinal Chemistry 112 (2024): 117894. 10.1016/j.bmc.2024.117894 39214013

[9] S. Ishida , J. Lee , D. J. Thiele , and I. Herskowitz , “Uptake of the Anticancer Drug Cisplatin Mediated by the Copper Transporter Ctr1 in Yeast and Mammals,” Proceedings of the National Academy of Sciences 99 (2002): 14298. 10.1073/pnas.162491399 PMC137878 12370430

[10] H. Muley , R. Fadó , R. Rodríguez‐Rodríguez , and N. Casals , “Drug Uptake‐Based Chemoresistance in Breast Cancer Treatment,” Biochemical Pharmacology 177 (2020): 113959. 10.1016/j.bcp.2020.113959 32272110

[11] R. Ali , M. Aouida , A. A. Sulaiman , S. Madhusudan , and D. Ramotar , “Can Cisplatin Therapy Be Improved? Pathways That Can Be Targeted,” International Journal of Molecular Sciences 23 (2022): 7241. 10.3390/ijms23137241 PMC9266583 35806243

[12] A. Sajid , H. Rahman , and S. V. Ambudkar , “Advances in the Structure, Mechanism and Targeting of Chemoresistance‐Linked ABC Transporters,” Nature Reviews Cancer 23 (2023): 762. 10.1038/s41568-023-00612-3 37714963

[13] G. Housman , S. Byler , S. Heerboth , et al., “Drug Resistance in Cancer: An Overview,” Cancers 6 (2014): 1769. 10.3390/cancers6031769 PMC4190567 25198391

[14] T. Harayama and H. Riezman , “Understanding the Diversity of Membrane Lipid Composition,” Nature Reviews Molecular Cell Biology 19 (2018): 281. 10.1038/nrm.2017.138 29410529

[15] W. Szlasa , I. Zendran , A. Zalesińska , M. Tarek , and J. Kulbacka , “Lipid Composition of the Cancer Cell Membrane,” Journal of Bioenergetics and Biomembranes 52 (2020): 321. 10.1007/s10863-020-09846-4 PMC7520422 32715369

[16] H. J. C. Berendsen , D. van der Spoel , and R. van Drunen , “GROMACS: A Message‐Passing Parallel Molecular Dynamics Implementation,” Computer Physics Communications 91 (1995): 43.

[17] J. S. Hub , B. L. de Groot , and D. van der Spoel , “g_wham─A Free Weighted Histogram Analysis Implementation Including Robust Error and Autocorrelation Estimates,” Journal of Chemical Theory and Computation 6 (2010): 3713.

[18] C. Zazza , G. Mancini , G. Brancato , and V. Barone , “ *In Silico* Study of Molecular‐Engineered Nanodevices: A Lockable Light‐Driven Motor in Dichloromethane Solution,” The Journal of Physical Chemistry Letters 4 (2013): 3885.

[19] C. Zazza , N. Sanna , S. Borocci , and F. Grandinetti , “A Computational Study of a [2]Rotaxane Molecular Shuttle with All‐Atoms Molecular Dynamics and Density Functional Theory Simulations in Solution,” ChemPhysChem 27 (2025): e202500660. 10.1002/cphc.202500660 PMC12810620 41252349

[20] T. Lu , “Visualization Analysis of Covalent and Noncovalent Interactions in Real Space,” Angewandte Chemie International Edition 64 (2025): e202504895. 10.1002/anie.202504895 40323713

[21] S. Borocci , F. Grandinetti , N. Sanna , and C. Zazza , “A Theoretical Investigation on the Hydrogen Bond Based on the GLED Method of Bonding Analysis,” Journal of Computational Chemistry 47 (2026): e70348. 10.1002/jcc.70348 PMC12969774 41800755

[22] S. Borocci , F. Grandinetti , N. Sanna , and C. Zazza , “Assaying the Nature of the Chemical Bond by Visualizing and Counting the Electrons with Negative Energies,” The Journal of Chemical Physics 163 (2025): 034107. 10.1063/5.0262841 40662705

[23] C. Zazza , F. Grandinetti , N. Sanna , S. Borocci , and H. S. Rzepa , “Direct Evidence for a Carbon–Carbon One‐Electron σ‐Bond, or a Weak Carbon–Carbon Interaction?,” ChemistryOpen 15 (2026): e70242. 10.1002/open.70242 PMC13244567 42257369

[24] E. R. Almeida , P. V. Z. C. Goliatt , H. F. Dos Santos , and F. Picaud , “Translocation Processes of Pt(II)‐Based Drugs through Human Breast Cancer Cell Membrane: *In* *Silico* Experiments,” Journal of Chemical Information and Modeling 63 (2023): 6141. 10.1021/acs.jcim.3c00943 37751589

[25] D. Belletto , S. Scoditti , S. Borocci , N. Sanna , C. Zazza , and E. Sicilia , “Molecular Dynamics Simulation of Passive Diffusion across a Human Breast Cancer Cell Membrane Model. Comparison between Cisplatin and Its Pt(IV) Derivatives,” Journal of Chemical Information and Modeling 66 (2026): 3150. 10.1021/acs.jcim.5c02819 PMC13014451 41787885

[26] T. Lu and Q. Chen , “Independent Gradient Model Based on Hirshfeld Partition: A New Method for Visual Study of Interactions in Chemical Systems,” Journal of Computational Chemistry 43 (2022): 539. 10.1002/jcc.26812 35108407

[27] P. Wu , R. Chaudret , X. Hu , and W. Yang , “Noncovalent Interaction Analysis in Fluctuating Environments,” Journal of Chemical Theory and Computation 9 (2013): 2226. 10.1021/ct4001087 PMC3720145 23894230

[28] T. Lu and Q. Chen , “Visualization Analysis of Weak Interactions in Chemical Systems,” in Comprehensive Computational Chemistry, (Elsevier, 2023), 240–264.

[29] T. Lu , “A Comprehensive Electron Wavefunction Analysis Toolbox for Chemists, Multiwfn,” The Journal of Chemical Physics 161 (2024): 082503. 10.1063/5.0216272 39189657

[30] M. Cossi , N. Rega , G. Scalmani , and V. Barone , “Energies, Structures, and Electronic Properties of Molecules in Solution with the C‐PCM Solvation Model,” Journal of Computational Chemistry 24 (2003): 669, We have introduced a static (or zero‐frequency, *Ɛ*) dielectric constant value of 4 for modelling the internal region of the biological membrane. 10.1002/jcc.10189 12666158

[31] A. D. Becke , “Density‐Functional Exchange‐Energy Approximation with Correct Asymptotic‐Behavior,” Physical Review A 38 (1988): 3098. 10.1103/physreva.38.3098 9900728

[32] T. Yanai , D. P. Tew , and N. C. Handy , “A New Hybrid Exchange‐Correlation Functional Using the Coulomb‐Attenuating Method (CAM‐B3LYP),” Chemical Physics Letters 393 (2004): 51.

[33] S. Grimme , J. Antony , S. Ehrlich , and H. Krieg , “A Consistent and Accurate Ab‐Initio Parameterization of Density Functional Dispersion Correction (DFT‐D) for the 94 Elements H‐Pu,” The Journal of Chemical Physics 132 (2010): 154104. 10.1063/1.3382344 20423165

[34] E. R. Johnson and A. D. Becke , “A Post‐Hartree‐Fock Model of Intermolecular Interactions: Inclusion of Higher‐Order Corrections,” The Journal of Chemical Physics 124 (2006): 174104. 10.1063/1.2190220 16689564

[35] M. J. Frisch , G. W. Trucks , H. B. Schgel , et al., Gaussian 16, Revision C.01, (Gaussian, Inc., 2016).

[36] R. F. W. Bader , “A Quantum‐Theory of Molecular‐Structure and Its Applications,” Chemical Reviews 91 (1991): 893.

[37] R. F. W. Bader , “Bond Paths Are Not Chemical Bonds,” The Journal of Physical Chemistry A 113 (2009): 10391. 10.1021/jp906341r 19722600

[38] A ‘‘critical point’’ (CP, ** *r* ** _ **cp** _) in the *ρ*(** *r* **) electron density is a particular point in space in which the first derivatives of the density vanish (i.e., *∇ρ*(*r*) = ** *0* **, where each individual derivative in the gradient operator, *∇*, is zero and not just their sum). The rank of a CP is equal to the number of non‐zero eigenvalues of the symmetric matrix known as the Hessian of *ρ*(** *r* ** _ **cp** _) [non‐zero curvatures of *ρ*(** *r* ** _ **cp** _) at the CPs] and signature is the algebraic sum of the signs of eigenvalues. There are then four possible values for CPs of rank three. They are (3, −3), (3, −1), (3, +1) and (3, +3). In the case of (3, −3), all curvatures are negative and *ρ* is a local maximum at ** *r* ** _ **cp** _. For (3, −1), the two curvatures are negative and *ρ* is a maximum at * **r** * ** _cp_ ** lying in the plane defined by their local corresponding axes. Further, *ρ* is a minimum at CP along the third axis, which is perpendicular to this plane. The (3, +1) CP has two positive curvatures and *ρ* is a minimum at CP in the plane defined by their corresponding axes. Further, *ρ* is a maximum at CP along the third axis, which is perpendicular to this plane. The (3, +3) CP represents all curvatures are positive and *ρ* is a local minimum at * **r** * _ **cp** _ (cage critical points, CCPs). The topological distribution of the *ρ*(** *r* **) is able to characterize the nuclear positions featuring * **r** * _ **cp** _ of kind (3, −3) since the nuclei act as the attractors of the gradient of *ρ* distribution of molecules. The presence of a (3, −1) CP and related atomic interaction line highlights that electronic charge density is accumulated between the nuclei that are linked by a chemical bond. The (3, +1) CP are typically found in the interior of the ring and are known as ring critical points (RCPs).

[39] C. Zazza , N. Sanna , S. Borocci , and F. Grandinetti , “High‐Performance Epothilone‐Based Microtubule Stabilizers as Anticancer Agents: Insights from DFT/QTAIM/MD Computational Modeling,” ChemistrySelect 10 (2025): e02546.

[40] C. Zazza , Comments on “Benzimidazo[1,2‐c]Quinazolines Luminescent Materials: Synthesis, Molecular Packing, and Aggregation Effects,” ChemPlusChem 91 (2026): e202500682. 10.1002/cplu.202500682 41905813

[41] C. Zazza , N. Sanna , S. Borocci , and F. Grandinetti , “QTAIM Analysis of a [2]Rotaxane Molecular Shuttle with a 2,2′‐Bipyridyl Rigid Core,” ChemPhysChem 26 (2025): e202500074. 10.1002/cphc.202500074 PMC12188175 40178147

[42] C. Zazza , N. Sanna , and F. Grandinetti , “Oxoaporphine‐Metformin Rhodium(III) Hybrid Ionic Complex With Antidiabetes and Anticancer Effects: New Insights From DFT Computations,” International Journal of Quantum Chemistry 126 (2026): e70244.

[43] G. Gramse , A. Dols‐Perez , M. A. Edwards , L. Fumagalli , and G. Gomila , “Nanoscale Measurement of the Dielectric Constant of Supported Lipid Bilayers in Aqueous Solutions with Electrostatic Force Microscopy,” Biophysical Journal 104 (2013): 1257. 10.1016/j.bpj.2013.02.011 PMC3602784 23528085

[44] A. Melchior , J. M. Martínez , R. R. Pappalardo , and E. S. Marcos , “Hydration of Cisplatin Studied by an Effective Ab Initio Pair Potential Including Solute–Solvent Polarization,” Journal of Chemical Theory and Computation 9 (2013): 4562. 10.1021/ct400433c 26589171

[45] A. Melchior , M. Tolazzi , J. M. Martínez , R. R. Pappalardo , and E. S. Marcos , “Hydration of Two Cisplatin Aqua‐Derivatives Studied by Quantum Mechanics and Molecular Dynamics Simulations,” Journal of Chemical Theory and Computation 11 (2015): 1735. 10.1021/ct500975a 26574384

[46] Radial Distribution Functions (RDF) for Pt(II)‐Ow contacts is bulk water reports maximum values lying in the range between 3.98 and 3.51 Å [44, 45]. The same analysis reports—taking into account an equilibrated 30 ns MD simulations in the middle of the model membrane imposed [24]—a Pt(II)‐OW RDF with a maximum at ~2.9 Å [25].

