# Supplementary material for: On the Dynamics of Noncovalent Interactions Modulating Passive Diffusion of Cisplatin Across a Human Breast Cancer Cell Membrane: An amIGM + GLED View
Source: Chempluschem. 2026 Oct 13;91(10):e70258. doi: 10.1002/cplu.70258 (PMC13661178; doi:10.1002/cplu.70258)
Supplement: Supplementary file 1 [file CPLU-91-e70258-s001.pdf]

Electronic Supporting Information (ESI)

On the Dynamics of Non-Covalent Interactions Modulating Passive Diffusion of Cisplatin across a Human Breast Cancer Cell Membrane: an amIGM+GLED View

Costantino Zazza,*[a] Daniele Belletto, [b] Stefano Scoditti, [c,d] Nico Sanna,[a],[e] Stefano Borocci,[a],[f] Emilia Sicilia[b]

[a] Dr. Costantino Zazza, Prof. Nico Sanna, Prof. Stefano Borocci,
Department for Innovation in Biological, Agro-food and Forest systems, Università della Tuscia (DIBAF), L.go dell'Università, s.n.c., 01100 Viterbo, Italy.
E-mail: costantino.zazza@unitus.it

[b] Dr. Daniele Belletto, Prof. Emilia Sicilia
Department of Chemistry and Chemical Technologies, Università della Calabria, Ponte P. Bucci, Arcavacata di Rende, Calabria 87036, Italy
Dr. Stefano Scoditti

[c] Donostia International Physics Center, Donostia 20018, Spain.

[d] Polimero eta Material Aurreratuak: Fisika, Kimika eta Teknologia, Kimika Fakultatea, Euskal Herriko Unibertsitatea UPV/EHU, Donostia-San Sebastian 20018, Spain;

[e] Prof. Nico Sanna
CNR-ISTP (Istituto per la Scienza e Tecnologia dei Plasmi), Via G. Amendola 122/D, 70126 Bari, Italy.

[f] Prof. Stefano Borocci
Istituto per i Sistemi Biologici del CNR (ISB), Sede di Roma - Meccanismi di Reazione c/o Dipartimento di Chimica, Sapienza Università di Roma,
P.le A. Moro 5, 00185, Rome, Italy

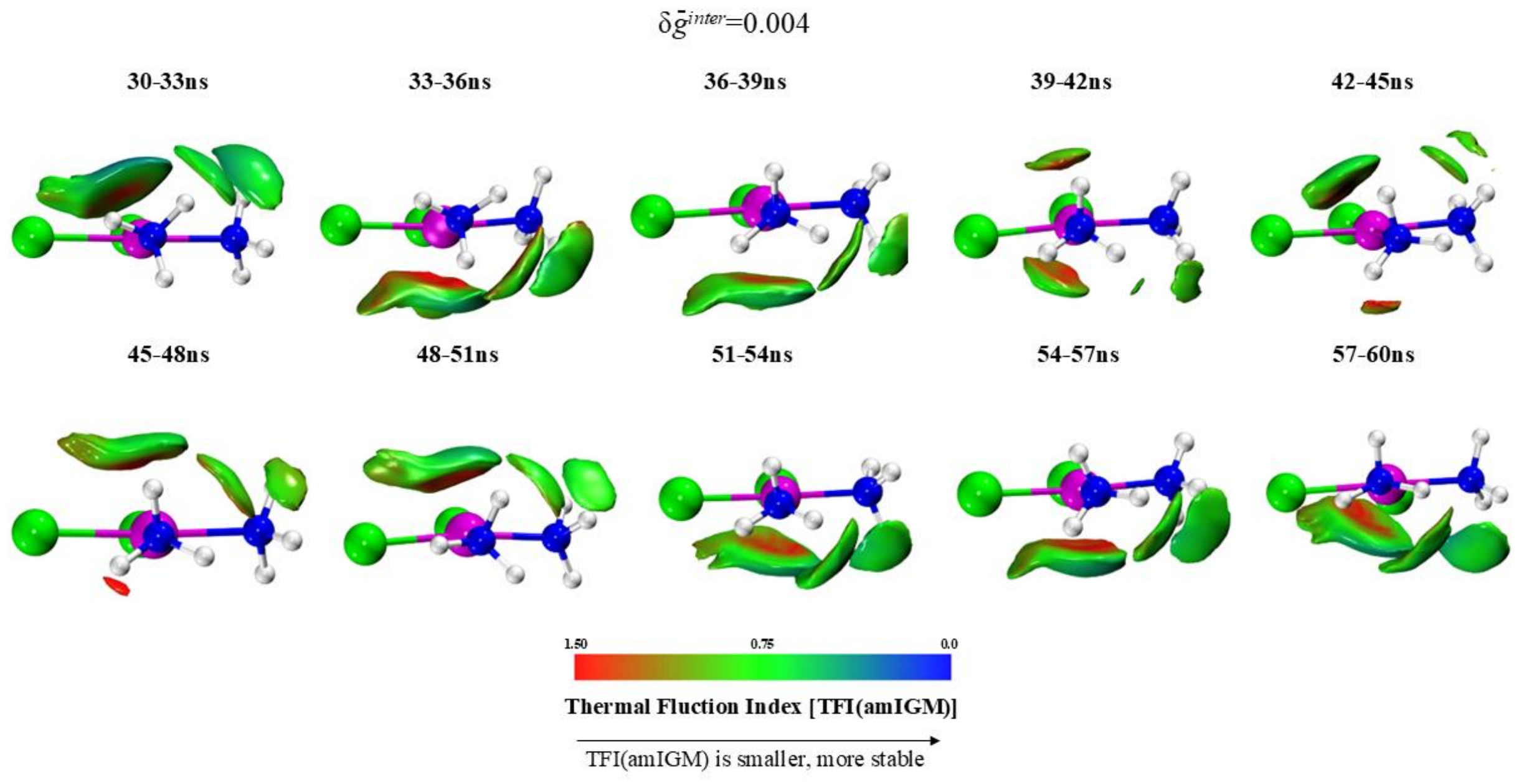

Figure S1: Thermal Fluctuation Index within the imposed amIGM modelling [TFI(amIGM; $\delta\bar{g}^{inter}$ = 0.004] as derived with a grid spacing of 0.12 a_0 and carried out considering ten MD trajectories of 3 ns each with the cisPt/wet drug in the middle point of a human breast cancer cell membrane model considered.

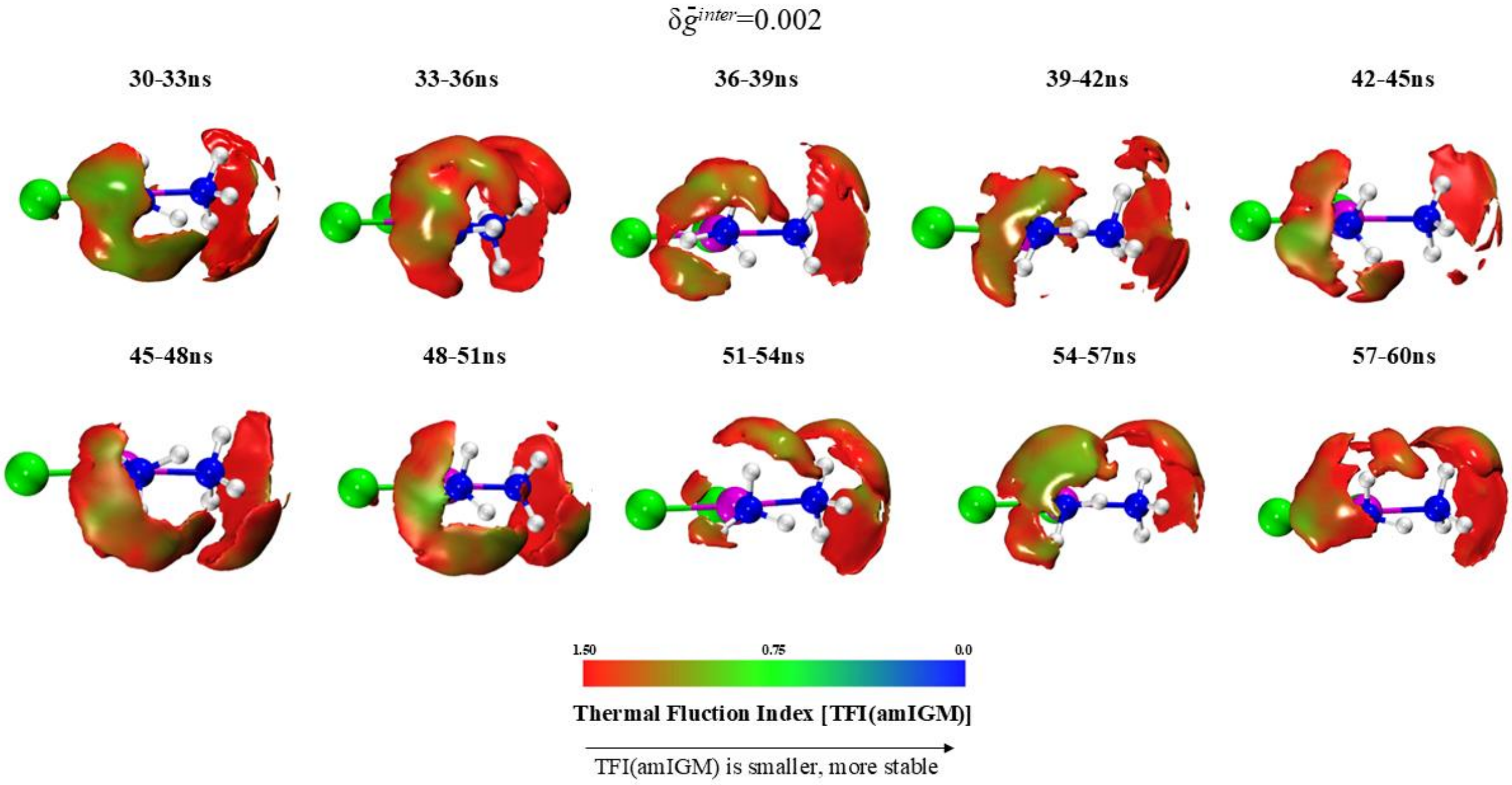

Figure S2: Thermal Fluctuation Index within the imposed amIGM modelling [TFI(amIGM; $\delta\bar{g}^{inter}$ = 0.002] as derived with a grid spacing of 0.12 a_0 and carried out considering ten MD trajectories of 3 ns each considering all the phospholipids of the membrane model in contact with the cisPt drug.

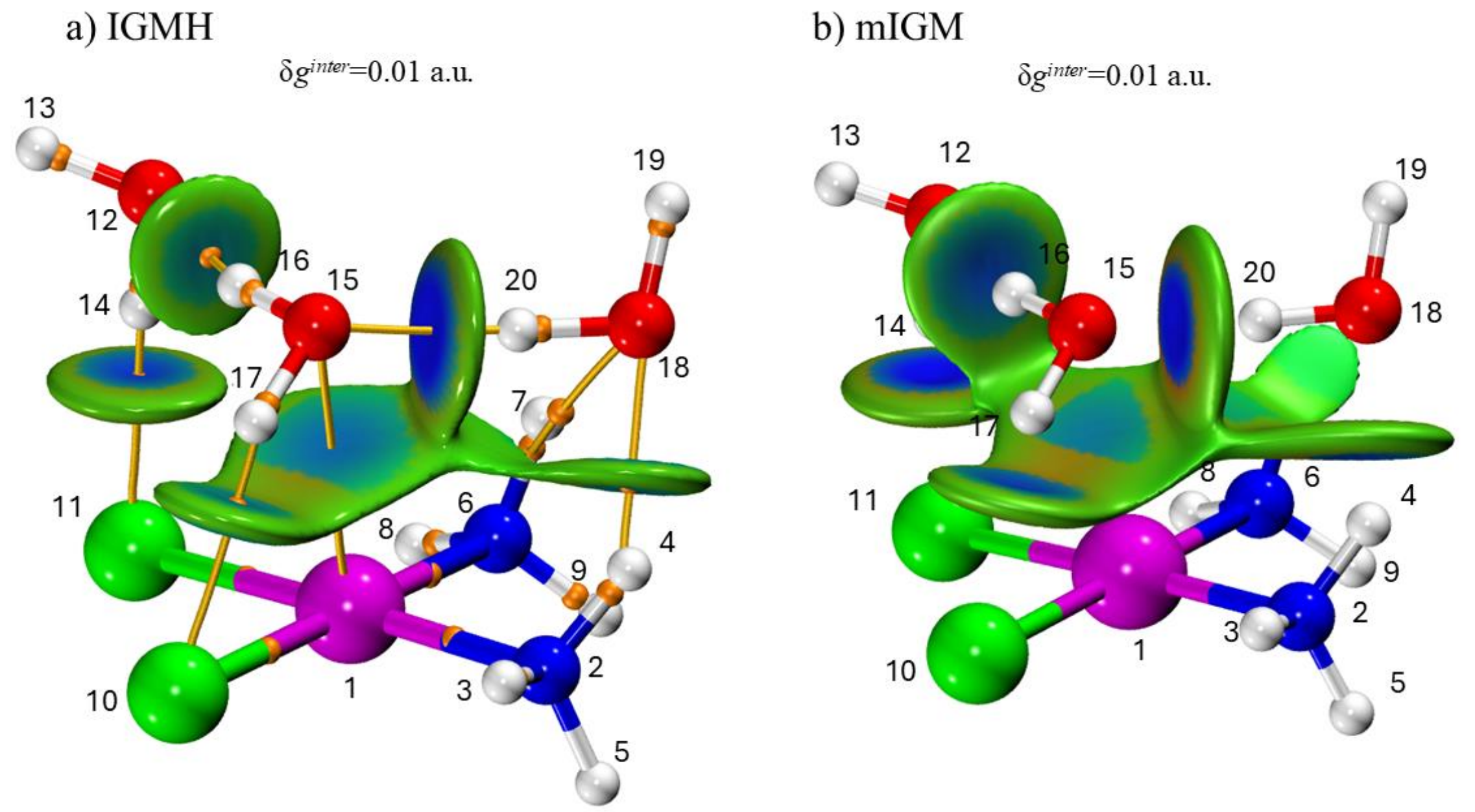

Figure S3: Comparison between IGMH and mIGM analysis imposing the same δg^{inter} isosurface and the same grid spacing of 0.12 a_0 for a selected MD frame showing three water molecules encircling the CisPt drug in the middle of the membrane model selected. The colour scheme applied reflects that reported in Fig.1 in the associated manuscript.
